# A Nonparametric Regression Approach to Control for Population Stratification in Rare Variant Association Studies

**DOI:** 10.1038/srep37444

**Published:** 2016-11-18

**Authors:** Qiuying Sha, Kui Zhang, Shuanglin Zhang

**Affiliations:** 1 Department of Mathematical Sciences, Michigan Technological University, Houghton, MI 49931, USA

## Abstract

Recently, there is increasing interest to detect associations between rare variants and complex traits. Rare variant association studies usually need large sample sizes due to the rarity of the variants, and large sample sizes typically require combining information from different geographic locations within and across countries. Although several statistical methods have been developed to control for population stratification in common variant association studies, these methods are not necessarily controlling for population stratification in rare variant association studies. Thus, new statistical methods that can control for population stratification in rare variant association studies are needed. In this article, we propose a principal component based nonparametric regression (PC-nonp) approach to control for population stratification in rare variant association studies. Our simulations show that the proposed PC-nonp can control for population stratification well in all scenarios, while existing methods cannot control for population stratification at least in some scenarios. Simulations also show that PC-nonp’s robustness to population stratification will not reduce power. Furthermore, we illustrate our proposed method by using whole genome sequencing data from genetic analysis workshop 18 (GAW18).

Recently, there is increasing interest to detect associations between rare variants and complex traits. The variant by variant methods used to detect associations of common variants may not be optimal for detecting associations of rare variants due to allelic heterogeneity as well as the extreme rarity of individual variants^1^. Many statistical methods for testing the association of rare variants have been developed by using joint information of multiple variants in a genomic region. These methods can be roughly divided into three groups: burden tests, quadratic tests, and combined tests.

Burden tests^1^,^2^,^3^,^4^,^5^ collapse rare variants in a genomic region into a single burden variable and then regress the phenotype on the burden variable to test for the cumulative effects of rare variants in the region^6^. Burden tests implicitly assume that all rare variants are causal and directions of effects are all the same. Quadratic tests include tests with statistics of quadratic form of score vector^7^,^8^,^9^ and also adaptive weighting methods^10^,^11^,^12^,^13^. Quadratic tests are robust to directions of effects of causal variants and are less affected by neutral variants than burden tests do. If most of the rare variants are causal and directions of effects of causal variants are all the same, burden tests can outperform quadratic tests; otherwise, quadratic tests perform better. Combined tests^6^,^14^ combine information from burden tests, quadratic tests, and possibly other tests aiming to have advantages of multiple tests and to increase the robustness of tests.

All the aforementioned methods are population-based methods for unrelated individuals. It has been long recognized that, for population-based association studies, population stratification can seriously confound association results^15^,^16^. For rare variants this problem can be more serious, because the spectrum of rare variations can be very different in different populations. In common variant association studies, several methods that use a set of genomic markers genotyped in the same samples have been developed to control for population stratification. These methods include genomic control (GC) approach^17^,^18^,^19^, principal component (PC) based linear regression (PC-linear) approach^20^, and mixed linear model (MLM) approach^21^,^22^ among others. GC approach adjusts the ordinary chi-square test statistic *X*^2^ to *X*^2^/*λ* and assumes *X*^2^/*λ* follows a chi-square distribution, where the inflation factor *λ* can be estimated using genotypes at genomic markers. PC-linear approach summarizes the genetic background or ancestry information through the PCs of genotypes at genomic markers. The PCs can be further used to eliminate the effect resulting from population stratification through linear regressions. MLM approach corrects for a wide range of sample structures by explicitly accounting for pairwise relatedness between individuals.

Although several methods for controlling for population stratification have been developed for common variants, it remains unclear whether these methods are equally effective for rare variants. Because rare variants have typically arisen recently, they tend to show greater geographic clustering or more latent subpopulations than common variants that are typically older. The more geographic clusters or latent subpopulations, the more difficult it will be to control for population stratification. Mathieson and McVean^23^ demonstrated that rare variants can show a stratification that is systematically different from common variants. They also demonstrated that the commonly used methods such as GC, PC-linear, and MLM to control for population stratification in common variant associations are not necessarily controlling for population stratification in rare variant associations. Zhang *et al*.^24^ showed that the use of PCs calculated from common variants were effective to control for population stratification in rare variant associations. Jiang *et al*.^25^ also found that the PC based methods performed quite well while GC often yielded lower power. Note that both studies of Zhang *et al*.^24^ and Jiang *et al*.^25^ did not explicitly model the spatial structure of populations in their simulation studies. Zhang *et al*.^24^ used two continental groups from the 1000 Genomes Project with six and four subpopulation groups, respectively. Jiang *et al*.^25^ simulated data with two populations. Lissgarten *et al*.^26^ reported that FaST-LMM Select (a MLM approach) could control for population stratification when samples were from spatially structured populations. However, their approach reduced power substantially when causal rare variants are spatially clustered^26^,^27^.

In this article, we propose a PC based nonparametric regression (PC-nonp) approach to control for population stratification in rare variant association studies. PC-nonp adjusts population effects of both trait values and genotypes at candidate loci for PCs of genotypes at genomic markers by applying nonparametric regressions. We use extensive simulation studies to evaluate the performance of the proposed method PC-nonp and compare the performance of PC-nonp with that of GC and PC-linear developed for common variants and recently proposed biased urn permutation test (BiasePerm)^28^ developed for rare variants. Simulation results show that PC-nonp can control for population stratification well in all scenarios while GC, PC-linear, and BiasedPerm cannot control for population stratification at least in some scenarios. Results also show that PC-nonp’s robustness to population stratification will not reduce power. Furthermore, we evaluate the performance of our approach by applying it to the whole genome sequencing data from genetic analysis workshop 18 (GAW18) and find that only PC-nonp is effective to control for population stratification.

## Method

**Figure 1:**
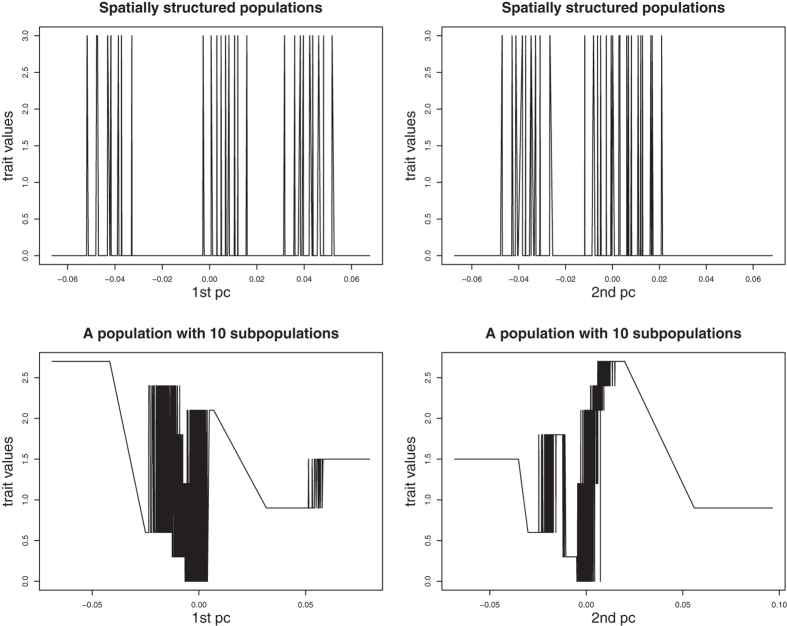
The relationships between the first two PCs of genotypes at 10,000 genomic markers and trait values. Genotypes at 10,000 genomic markers in the spatially structured populations are generated according to simulation set 2 in simulation section. Genotypes at 10,000 genomic markers in a population with 10 subpopulations are generated according to simulation set 1 in simulation section. The trait values in the spatially structured populations and in a population with 10 subpopulations are generated according to null distributions (without random error) in simulation set 2 and simulation set 1, respectively.

Consider a sample of *n* unrelated individuals. Suppose that each individual has been genotyped at a candidate locus (single variant or multiple variants) and at *L* genomic markers. Let *y*_*i*_, *x*_*i*_, and *p*_*i*_ denote the trait value, genotypic score at the candidate locus (weighted sum of genotypic scores if there are multiple variants), and the first *k* PCs (rescaled to the interval [0, 1]) of genotypes at genomic markers of the *i*^*th*^ individual. The PCs of genotypes at genomic markers are good summary measures of ancestry or genetic background. PC-linear is probably the most popular method to control for population stratification. However, this method is based on linear combinations of PCs. Furthermore, recently developed BiasePerm^28^ is based on linear combinations of PCs on logistic scale if we use PCs as covariate vector^28^. The relationships between trait values and PCs can be highly nonlinear and population effects cannot be corrected by simply using linear functions^23^. Figure 1 shows the relationships between trait values and the first two PCs of genotypes at 10,000 genomic markers in two structured populations. This figure shows that the relationships between trait values and PCs are highly nonlinear and the forms of the relationships are different in different populations. When the relationships are highly nonlinear and the forms of relationships are unknown, we should use more flexible regression methods rather than use linear regression. Nonparametric regression is a very flexible regression method and it does not require the form of regression function.

In this article, we propose a PC based nonparametric regression (PC-nonp) approach that adjusts population effects of both trait values and genotypes at candidate loci for PCs of genotypes at genomic markers by applying nonparametric regressions. That is,


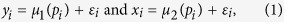


where *μ*_1_(**·**) and *μ*_2_(**·**) are regression functions with unknown forms and will be estimated using smoothing techniques. Let 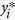 and 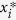 be the residuals of the nonparametric regressions. We can consider 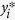 and 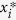 as the trait value and genotypic score at the candidate locus of the *i*^*th*^ individual after adjusting for population effects. We can construct association tests based on the residuals.

Many methods have been developed to estimate the unknown regression function, including local linear method^29^,^30^,^31^, kernel smoothing method^32^,^33^ and wavelet method^34^,^35^. We propose to use kernel smoothing method. Let *K*(**·**) be a kernel function with mode at 0. The kernel estimators of *μ*_1_(*p*_*i*_) and *μ*_2_(*p*_*i*_) are given by


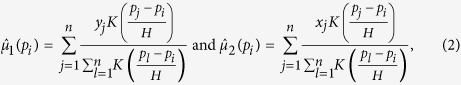


respectively, where *p*_*j*_ = (*p*_*j*1_, …, *p*_*jk*_) is the first *k* PCs for the *j*^*th*^ individual, *H* = (*h*_1_, …, *h*_*k*_) is the smoothing parameter, and 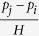 = 
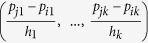
. If we denote 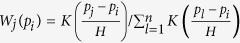, then 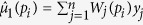 and 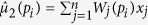. With these nonparametric estimators, the fitted values of trait and the fitted values of genotypic scores at the candidate locus are given by 
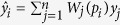
 and 
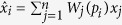
, respectively. Intuitively, 
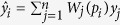
 and 
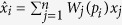
 are the weighted mean of trait values and weighted mean of genotypic scores of those individuals whose genetic background is similar to that of the *i*^*th*^ individual. Thus, we can consider residuals 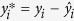 and 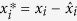 as the trait value and genotypic score of the *i*^*th*^ individual after adjusting for population stratification.

In this study, we use the quartic kernel^33^,


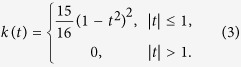


For computational consideration, we assume that *h*_1_ = ... = *h*_*k*_ = *h*. Then,


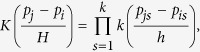


To test association between trait values and genotypes based on 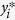 and 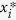, we can use score test with test statistic *T*_*score*_ = *U*^2^/*V*, where 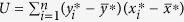 and 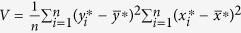. The statistic *T*_*score*_ asymptotically follows a chi-square distribution with one degree of freedom (df)^36^. For rare variants, *x*_*i*_ can be a weighted combination^2^ or collapsing^1^,^3^ of genotypes at multiple variants in a genomic region. Based on the residuals of the nonparametric regression, we can construct other rare variant association tests such as CMC^1^, SKAT^9^, and TOW^8^. We will discuss this issue in more details later in the discussion section. In this study, we use a single-variant test in which *x*_*i*_ is the genotypic score of a single variant and a regional test in which *x*_*i*_ is the weighted combination of genotypes at the variants in a genomic region^2^ to evaluate the performance of our proposed method.

We have so far assumed a given smoothing parameter in the kernel estimates. It is well known that choosing a proper value for smoothing parameter *h* is critical to kernel estimates of regression functions^32^,^37^. We use a method similar to that of Zhang *et al*.^35^ to choose smoothing parameter *h*. This method is based on the genotypes at a set of genomic markers. Suppose there are *L* genomic markers. We perform PC-nonp single-variant test for all the *L* genomic markers and denote *P*_1_, …, *P*_*L*_ as the associated *P*-values. If population stratification is well controlled for, *P*-values *P*_1_, …, *P*_*L*_ should follow a uniform distribution under the null hypothesis of no association. Let *F*_*n*_ be the empirical distribution function of the *P*-values *P*_1_, …, *P*_*L*_ and *F* be the uniform distribution function. The Kolmogorov test statistic 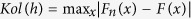 measures how close the distribution of the *P*-values *P*_1_, …, *P*_*L*_ and the uniform distribution are. We propose to choose h^*^ that minimizes the Kolmogorov test statistic, i.e.,


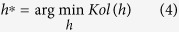


as the value of the smoothing parameter. h^*^ can be obtained by a simple grid search across a range of *h*. We divide the interval [0, ∞)into subintervals 0 ≤ *h*_1_ < … < *h*_*S*−1_ < *h*_*S*_ < ∞. Then, 
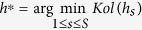
. The computational time to find h* increases linearly with *S*. However, h* needs to be calculated only once. We can use this h* to calculate the residuals of the nonparametric regression for trait values and genotypes at each variant. Let *k* denote the number of PCs used. In this study, we use *h*_*s*_ = 2^2(*s*−23)/(5+*k*)^, where *s* = 1, …, 30 and *k* = 10. It is worth noting that the smoothing parameter *h* is chosen with the *P*-values of a single-variant test, whichever test is actually used in testing associations.

### Software

R code for implementing our proposed method is given at Shuanglin Zhang’s homepage http://www.math.mtu.edu/~shuzhang/software.html. The R code includes three functions: PCA, choose_OPT_SMP, and Resid_Nonp. PCA gives the first *k* principal components of genotypes at genomic markers. choose_OPT_SMP chooses the optimal value of smoothing parameter. Given the value of the smoothing parameter, Resid_Nonp calculates the residuals of trait values and genotypes at a candidate region by applying nonparametric regression for PCs of genotypes at genomic markers.

### Comparison of Tests

We compare the performance of the proposed test with that of the following four tests. (1) Uncorrected: this test is also based on the score test statistic 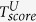. 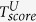 is the same as *T*_*score*_ but 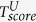 is based on the original trait values *y*_*i*_ and genotypic scores *x*_*i*_ instead of based on the residuals. (2) GC^17^: GC divides 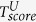 by an inflation factor *λ* and 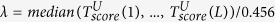, where 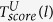 is the value of 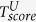 when 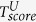 is applied to the *l*^*th*^ genomic marker. (3) PC-linear^20^: this test is the same as PC-nonp but PC-linear is based on the residuals of linear regression instead of based on the residuals of nonparametric regression. (4) The biased urn permutation test (BiasedPerm)^28^: in this permutation procedure, the odds of a subject being selected as a case are equal to his or her odds of disease conditional on confounder variables. In this study, PC-linear, PC-nonp, and BiasedPerm are based on the first 10 PCs of genotypes at the genomic markers.

### Simulations

We consider two sets of simulations: populations with *k*_0_ subpopulations and populations with spatially structured populations. In each set of simulations, we consider both qualitative and quantitative traits. To generate a qualitative disease affection status, we use a liability threshold model based on a continuous phenotype (quantitative trait). An individual is defined to be affected if the individual’s phenotype is at least one standard deviation larger than the phenotypic mean. This yields a prevalence of 16% for the simulated disease in the general population. In the following, we describe how to generate genotypes and how to generate a quantitative trait in the two sets of simulations.

#### Simulation Set 1: Populations with *k*_0_ Subpopulations

This set of simulations is based on allele frequencies at 24,487 variants calculated from the empirical Mini-Exome genotype data provided by the genetic analysis workshop 17 (GAW17). The genotypes of GAW17 data set are extracted from the sequence alignment files provided by the 1000 Genomes Project for their pilot3 study (http://www.1000genomes.org). GAW17 data contain genotypes of 697 unrelated individuals at 24,487 variants. The distributions of MAF at rare variants (MAF < 0.01) and MAF at common variants of 24,487 variants are given in Figure S1.

To generate genotypes of individuals in a population with *k*_0_ subpopulations, we follow Price *et al*.^20^, Ionita-Laza *et al*.^38^, and Qin *et al*.^39^. For each variant, we randomly select a variant from 24,487 variants and take the MAF at this variant as the ancestral population allele frequency *p*. Then, independently draw *k*_0_ values 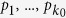 from a beta-distribution with parameters *p*(1 − *F*_*st*_)/*F*_*st*_ and (1 − *p*)(1 − *F*_*st*_)/*F*_*st*_, where *F*_*st*_ is the Wright’s measure of population subdivision^40^ (in this study, *F*_*st*_ = 0.01). For each variant, we accept 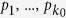 as allele frequencies for the *k*_0_ subpopulations if 
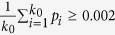
; we redraw 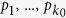 otherwise. The MAF distributions at the rare variants (*MAF* < 0.01) and at the common variants for *k*_0_ = 5 are given in Figure S1.

To evaluate type I error, we generate trait values independent of genotypes by using the model:


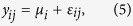


where *y*_*ij*_ denotes the trait value of the *j*^*th*^ individual in the *i*^*th*^ subpopulation, *ε*_*ij*_ follows a standard normal distribution, and *μ*_*i*_ is the population mean of the *i*^*th*^ subpopulation. In this study, if *k*_0_ ≤ 2, we set *μ*_1_ = 0 and *μ*_2_ = *μ*; otherwise, we set 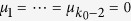 and 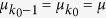, where *μ* = 5 if *k*_0_ = 20; otherwise *μ* = 2.

To evaluate power, we consider *n*_*T*_ variants (possibly both rare and common variants) in a genomic region. We randomly choose *n*_*c*_ from the *n*_*T*_ variants as causal variants (in this study, *n*_*c*_ = *n*_*T*_/2). For the *j*^*th*^ individual in the *i*^*th*^ subpopulation, let *x*_*ijl*_ denote the genotypic score of the *j*^*th*^ individual in the *i*^*th*^ subpopulation at the *l*^*th*^ causal variant. We assume that all the *n*_*c*_ causal variants have the same heritability such that rarer variants have larger effects. Under this assumption, the disease model is given by


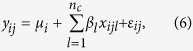


where *β*_*l*_ are constants and their values depend on the total heritability.

#### Simulation Set 2: Spatially Structured Populations

We generate genotypes and phenotypes under spatially structured populations using the methods similar to those of Mathieson and McVean^23^. Briefly, the space is divided into *K*_0_ × *K*_0_ grid squares. Then, we generate genotypes by starting with a number of individuals and their locations on the grid. We work backward in time to generate random genealogical events. Each event is either a coalescence of two lineages or a migration of a single lineage from one square to another. The relative rates of coalescence and migration depend on the population-scaled migration rate M and the number and distribution of lineages on the grid (see Supplement materials or Mathieson and McVean^23^ for details).

To generate quantitative traits under null hypothesis, let *ϕ*: |1, *n*| → |1, *K*_0_| × |1, *K*_0_| be a function that maps each individual to the grid square from which they originated. Then, we generate the trait value of the *i*^*th*^ individual by *y*_*i*_ = *βR*_*ϕ*(*i*)_ + *ε*_*i*_, where *ϕ*(*i*) = (*l, j*) if the *i*^*th*^ individual originates from grid square *l, j*; *R*_*l*,*j*_ is the nongenetic risk in grid square *l, j*; *ε*_*i*_ is a standard normal random number; and *β* is a constant. We use the following three models to determine the value of *R*_*l*,*j*_. Model 0: no population stratification in which *R*_*l*,*j*_ = 0 for all *l* and *j*. Model 1: a small and sharp spatial distribution in which *R*_*l*,*j*_ = 1 if *l*_0_ ≤ *l* ≤ *l*_0_ + 3 and *j*_0_ ≤ *j* ≤ *j*_0_ + 3 for *l*_0_ = *j*_0_ = 6, or 20 − *l*_0_ = *j*_0_ = 6, or *l*_0_ = *j*_0_ = 14; *R*_*l*,*j*_ = 0 otherwise. Model 2: a wide and smooth spatial distribution in which 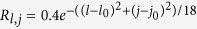 for *l*_0_ = *j*_0_ = 6. In this study, we use the following parameters: *K*_0_ = 20, *M* = 0.01, and *β* = 2.

Under alternative hypothesis, we assume that there are *n*_*T*_ variants in a genomic region. We randomly choose *n*_*c*_ from the *n*_*T*_ variants as causal variants. For an individual, let *x*_*l*_ denote the genotypic score at the *l*^*th*^ causal variant. Under the assumption that all the *n*_*c*_ causal variants have the same heritability, the trait value for an individual is generated by


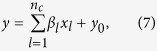


where *y*_0_ is the trait value generated under null hypothesis.

## Results

### Existence of the minimum of Kolmogorov test statistic *Kol*(*h*)

**Figure 2:**
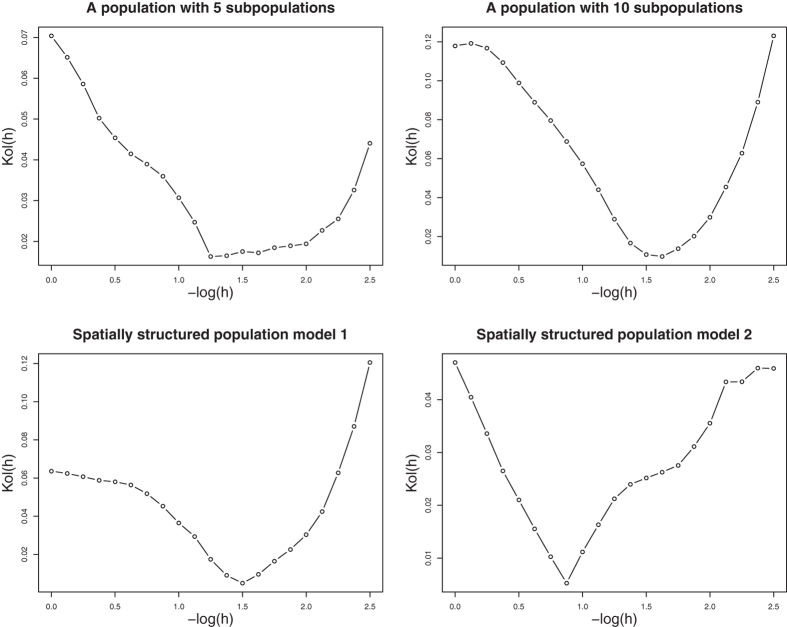
The relationships between −log(smoothing parameter *h*) and Kolmogorov test statistic Kol(*h*) in four structured populations.

We first perform simulation studies to evaluate the existence of the minimum of Kolmogorov test statistic *Kol*(*h*). We generate trait values and genotypes at 10,000 variants under simulation set 1 for *k*_0_ = 5 and *k*_0_ = 10 and under simulation set 2 models 1 and 2. Under each of the four scenarios, we calculate *Kol*(*h*) for different values of *h*. The relationships between *Kol*(*h*) and −log(*h*) under the four scenarios are given in Fig. 2. This figure shows that the curves of *Kol*(*h*) under the four scenarios are all bowl shaped and thus have minimum. The histograms of 10,000 *P*-values of the proposed test for different values of *h* are given in Figures S2–S5 for the four scenarios, respectively. From these figures, we can see that when *h* is large, population effects are not adjusted enough and thus the number of small P-values are more than expected; when *h* is small, population effects are over adjusted and thus the number of large *P*-values are more than expected; when *h* minimizes *Kol*(*h*), the distribution of *P*-values is very close to the uniform distribution.

### Evaluate type I error rates

We use 10,000 replicated samples to evaluate type I error rates. For BiasedPerm, we use 5,000 permutations to evaluate *P*-values. For all other tests, we use asymptotic distributions to evaluate *P*-values. For 10,000 replicated samples, the 95% confidence intervals (CIs) for type I error rates of nominal levels 0.01 and 0.001 are (0.008, 0.012) and (0.00037, 0.00163), respectively.

To evaluate type I error rates, we first want to see the performance of the asymptotic distributions we used. For this purpose, we perform simulations under null hypothesis in a homogenous population (*k*_0_ = 1 in simulation set 1) and in the case of no population stratification (model 0 in simulation set 2). Type I error rates are given in Tables 1 and 2 for quantitative traits and qualitative traits, respectively. Table 1 shows that, for quantitative traits, type I error rates of all the four tests in all the scenarios are within the corresponding 95% confidence intervals, which indicates that the asymptotic distributions work very well. Table 2 shows that, for qualitative traits, most of the type I error rates are within the corresponding 95% CIs and those of the type I error rates that are not in the 95% CIs are very close to the corresponding 95% CIs, which indicates that the asymptotic distributions approximately work well.

Type I error rates under structured populations in simulation set 1 for *k*_0_ = 2, 10, 20 are given in Tables 3 and 4 for quantitative traits and qualitative traits, respectively. As shown by these two tables, Uncorrected has inflated type I error rates in all the scenarios. GC cannot control for population stratification for quantitative traits when *k*_0_ = 10 and 20 because most variants have very small correlation with the trait. PC-linear and BiasedPerm cannot control for population stratification when *k*_0_ = 20 because the linear combinations of the first 10 PCs cannot discriminate 20 subpopulations. Only PC-nonp can control for population stratification in all simulation scenarios. If we increase the number of PCs, PC-linear and BiasedPerm may control for population stratification when *k*_0_ = 20. The problems to use PC-linear and BiasedPerm to control for population stratification are (1) we do not know how many PCs should be used and (2) increasing the number of PCs may decrease the power.

Type I error rates under spatially structured populations in simulation set 2 for models 1 and 2 are given in Tables 5 and 6 for quantitative traits and qualitative traits, respectively. These two tables show that Uncorrected has inflated type I error rates in all the scenarios. GC cannot control for population stratification for single variant test because most variants have very small correlation with the trait. PC-linear and BiasedPerm have inflated type I error rates under model 1 because these two methods try to correct highly nonlinear relationships on the basis of linear functions of relatedness. PC-nonp can control for population stratification well in all simulation scenarios because nonparametric regressions can adapt any function, linear or nonlinear.

### Power comparison

**Figure 3:**
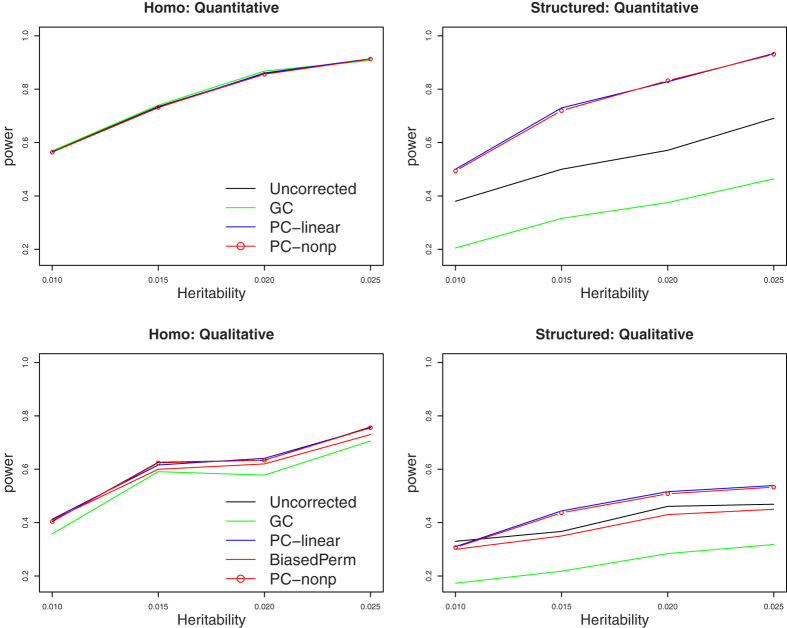
Power comparisons based on populations with k_0_ subpopulations. “Homo” means that simulations are based on a homogenous population (*k*_0_ = 1 in simulation set 1). “Structured” means that simulations are based on a structured population with 10 subpopulations.

To evaluate if PC-nonp’s robustness to population stratification will reduce power, we perform simulation studies to compare power using regional tests under *k*_0_ = 1 and *k*_0_ = 10 in simulation set 1 and under models 0 and 2 in simulation set 2, in which all tests except Uncorrected can control for population stratification well. Power comparisons under *k*_0_ = 1 and *k*_0_ = 10 in simulation set 1 are given in Fig. 3. This figure shows that, when there is no population stratification (a homogenous population), all tests have very similar powers. When there is population stratification (a structured population with 10 subpopulations), PC-nonp and PC-linear are more powerful than Uncorrected and BiasedPerm, and GC has the lowest power. GC loses power because it has a larger inflation factor when there is population stratification. BiasedPerm essentially performs permutation within subpopulations and thus it will lose power when there are a large number of subpopulations. Uncorrected loses power because, in the structured population with 10 subpopulations, different trait value means in subpopulations weaken the association signal. PC-nonp and PC-linear do not lose power because, after adjusted for population effects, it appears that PC-nonp and PC-linear perform association tests in a homogenous population.

**Figure 4:**
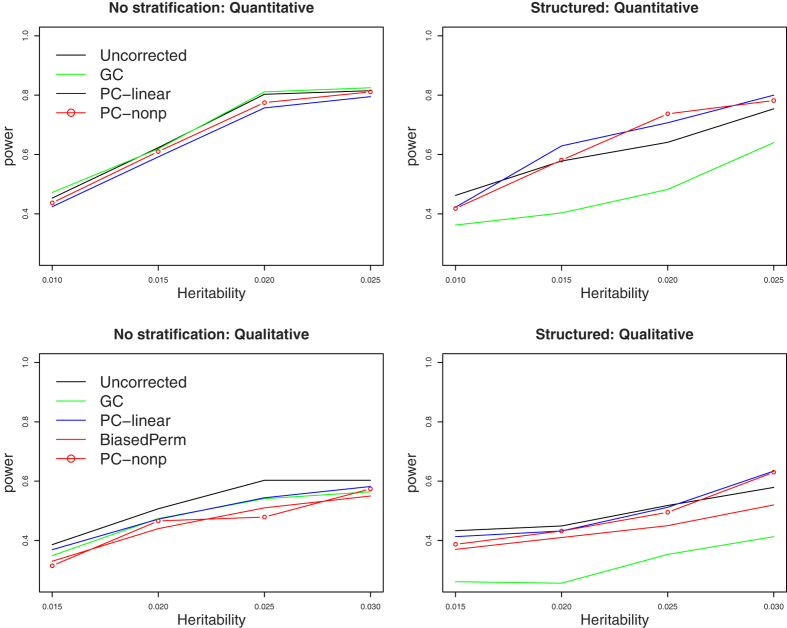
Power comparisons based on spatially structured populations. “No stratification” means that trait values have no relation with spatial position (model 0 in simulation set 1). “Structured” means that trait values are generated according to spatially structured model 2.

Power comparisons under models 0 and 2 in simulation set 2 are given in Fig. 4. As shown by this figure, for quantitative traits, the pattern of power comparisons is very similar to that in Fig. 3. For qualitative traits, Uncorrected is the most powerful one. The pattern of power comparisons among PC-nonp, PC-linear, BiasedPerm, and GC is very similar to that in Fig. 3.

### Analysis of GAW18 whole genome sequencing data set

The data set for GAW18 includes whole genome sequencing (WGS) data of 959 individuals (464 directly sequenced and the rest imputed) from 20 Mexican American pedigrees from San Antonio, Texas. There are 21–76 individuals in each pedigree. Phenotype data include sex, age, year of examination, systolic and diastolic blood pressure (SBP and DBP), use of antihypertensive medications, and tobacco smoking at up to four time points.

**Figure 5:**
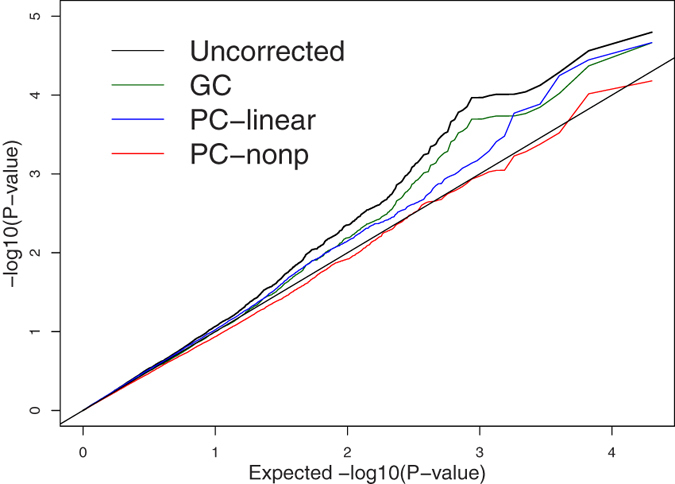
Quantile-quantile plots of observed −log10(P-values) of four tests and expected −log10(P-value). All quantile-quantile plots are averaged over 4 times replicated draws in order to show the average effect. Each draw, we randomly choose 10,000 SNPs without overlap from the 41,754 uncommon SNPs with MAF between 0.02 and 0.05 on chromosome 17.

Since Mexican American population is admixture population, association studies based on unrelated individuals from this population may be subjected to bias due to population stratification. For our purpose, we extract 132 genetically unrelated individuals from the 20 pedigrees with phenotypes and WGS data and select SBP as the trait of interest while take sex, age, use of antihypertensive medications, and tobacco smoking as covariates. For WGS data, we only consider one chromosome (chromosome 17). Among the 132 unrelated individuals, there are 404,032 SNPs on chromosome 17. Since the sample size is small, we only consider the 41,754 uncommon SNPs with MAF between 0.02 and 0.05 instead of including rare SNPs. We randomly draw 10,000 SNPs from the 41,754 SNPs without replacement and test association between the phenotype and each of the 10,000 SNPs using each of the four tests: Uncorrected, GC, PC-linear, and PC-nonp. We repeat the drawing procedure 4 times with re-drawing 10,000 SNPs from the 41,754 SNPs. Quantile-quantile plots of the observed −log_10_(*P*-*values*) of the four tests and expected log_10_(*P*-*value*) under the assumption of uniform distribution of P-values are given in Fig. 5. All quantile-quantile plots are averaged over 4 draws in order to show the average effect. Since we randomly draw 10,000 SNPs across chromosome 17, it is unlikely that there are a large number of SNPs in the 10,000 SNPs associated with SBP. Therefore, if population stratification can be well controlled for, P-values should proximately follow a uniform distribution. Figure 5 shows that only P-values of PC-nonp nearly follow a uniform distribution while for all other tests, the number of small P-values is more than expected.

## Discussion

With the development of next-generation sequencing technology, there is increasing interest to detect associations between rare variants and complex traits. Many statistical methods have been developed for detecting rare variant associations. However, these methods may be subject to bias due to population stratification and, as pointed out by Mathieson and McVean^23^, existing methods developed to control for stratification are not necessarily effective in rare variant associations. Therefore, statistical methods that can control for population stratification in rare variant association studies are needed. In this article, we propose the PC-nonp approach to control for population stratification in rare variant association studies. To apply PC-nonp, we first calculate PCs of genotypes at the genomic markers. Then, we use these PCs to adjust population effects of both trait values and genotypes at a candidate locus by applying nonparametric regressions. Our simulations show that the proposed PC-nonp can control for population stratification well in all scenarios while existing methods cannot control for population stratification at least in some scenarios. Simulations also show that PC-nonp’s robustness to population stratification will not reduce power. Applications to the GAW18 whole genome sequencing data set also show that our proposed method can control for population stratification better than existing methods.

Although we describe our proposed method using a single-variant test and a weighted sum regional test, our method can be applied to most existing rare variant association tests such as CMC^1^, SKAT^9^, and TOW^8^. To apply our method to SKAT and TOW, denote *y*_*i*_ and *x*_*im*_ as the trait value and genotypic score at the *m*^*th*^ variant of the *i*^*th*^ individual. Let 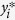 and 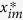 denote the residuals of nonparametric regressions *y*_*i*_ = *μ*(*p*_*i*_) + *ε*_*i*_ and *x*_*im*_ = *μ*_*m*_(*p*_*i*_) + *ε*_*im*_, where *i* = 1, …, *n* and *m* = 1, …, *M*. Based on the residuals 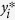 and 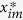, the test statistics of both SKAT and TOW can be written as 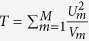, where 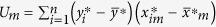. In TOW, 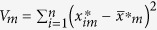 while, in SKAT, 
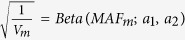
, the beta distribution density function with pre-specified parameters *a*_1_ and *a*_2_ evaluated at the sample MAF for the *m*^*th*^ variant in the data. To apply our method to CMC, suppose that M variants can be classified as *S*_*r*_ groups of rare variants and *S*_*c*_ individual variant sites. Define indicator variables *x*_*is*_(*i* = 1, …, *n*; *s* = 1, …, *S*_*r*_) for all individuals and the *S*_*r*_ groups of rare variants, where *x*_*is*_ = 1 if minor alleles at any variant in the *s*^*th*^ group of the *i*^*th*^ individual are present; *x*_*is*_ = 0 otherwise. Let *S* = *S*_*r*_ + *S*_*c*_ and define 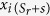
(*s* = 1, …, *S*_*c*_) as the genotypic score of the *i*^*th*^ individual at the *s*^*th*^ individual variant site. Let 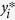 and 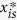 denote the residuals of nonparametric regressions *y*_*i*_ = *μ*(*p*_*i*_) + *ε*_*i*_ and *x*_*is*_ = *μ*_*s*_(*p*_*i*_) + *ε*_*is*_, where *i* = 1, …, *n* and *s* = 1, …, *S*. Based on residuals 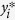 and 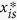, we cannot use *T*^2^ test because 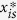 are not 0 and 1. We can use a score test or the improved score test^36^.

Zhang *et al*.^41^ proposed a semi-parametric test for association (SPTA) to control for population stratification. SPTA models the relationship between trait values, genotypic scores at the candidate marker, and PCs of genotypes at genomic markers through a semi-parametric model, where the exact form of relationship between trait values and PCs is assumed unknown, but trait values have linear relationship with genotypic scores at the candidate marker. Although SPTA and PC-nonp are equivalent for single-variant tests under quantitative traits, SPTA is difficult to extend to regional rare variant association tests such as SKAT and TOW because it is designed for single-variant tests.

## Additional Information

**How to cite this article**: Sha, Q. *et al*. A Nonparametric Regression Approach to Control for Population Stratification in Rare Variant Association Studies. *Sci. Rep.* **6**, 37444; doi: 10.1038/srep37444 (2016).

**Publisher’s note:** Springer Nature remains neutral with regard to jurisdictional claims in published maps and institutional affiliations.

## Supplementary Material

Supplemental Materials

## Figures and Tables

**Table 1:** Type I error rates of four tests in a homogenous population and in the case of no population stratification for quantitative traits.

Population Structures	Test	Alpha	Un-corrected	GC	PC-linear	PC-nonp
A Homogenous Population	SingleVariant	0.01	0.01054	0.01104	0.01096	0.01106
		0.001	0.00106	0.00124	0.0011	0.00114
	Regional	0.01	0.0103	0.01112	0.01078	0.01048
		0.001	0.00086	0.00112	0.00088	0.00096
No Population Stratification	SingleVariant	0.01	0.0105	0.00984	0.01254	0.00994
		0.001	0.0008	0.00068	0.00112	0.00076
	Regional	0.01	0.00942	0.00974	0.00936	0.0096
		0.001	0.00074	0.00078	0.00076	0.00072

Note: “A homogenous population” means *k*_0_ = 1 in simulation set 1; “no population stratification” means model 0 in simulation set 2.

**Table 2:** Type I error rates of four tests in a homogenous population and in the case of no population stratification for qualitative traits.

Population Structures	Test	Alpha	Un-corrected	GC	PC-linear	PC-nonp
A Homogenous Population	SingleVariant	0.01	0.0131	0.0076	0.0129	0.01178
		0.001	0.00168	0.00112	0.00182	0.00172
	Regional	0.01	0.00992	0.00958	0.00992	0.0121
		0.001	0.00124	0.00116	0.00122	0.00162
No Population Stratification	SingleVariant	0.01	0.01082	0.0053	0.01	0.0079
		0.001	0.00169	0.0007	0.00162	0.0014
	Regional	0.01	0.0113	0.00904	0.01176	0.00926
		0.001	0.00178	0.00138	0.00166	0.00134

Note: “A homogenous population” means *k*_0_ = 1 in simulation set 1; “no population stratification” means model 0 in simulation set 2.

**Table 3:** Type I error rates of four tests based on simulation set 1 for quantitative traits.

Number of Subpopulations	Test	Alpha	Un-corrected	GC	PC-linear	PC-nonp
2	SingleVariant	0.01	0.1516	0.00414	0.01026	0.01038
		0.001	0.05998	0.00032	0.00108	0.001
	Regional	0.01	0.258	0.0081	0.00995	0.00925
		0.001	0.1489	0.0004	0.00115	0.00095
10	SingleVariant	0.01	0.0503	0.01498	0.01096	0.01068
		0.001	0.01606	0.0026	0.0012	0.00108
	Regional	0.01	0.0549	0.0101	0.0107	0.0102
		0.001	0.0139	0.00085	0.0008	0.001
20	SingleVariant	0.01	0.04896	0.02602	0.04234	0.01004
		0.001	0.0214	0.0102	0.01632	0.00082
	Regional	0.01	0.0488	0.01285	0.0391	0.011
		0.001	0.0137	0.00255	0.0096	0.00075

**Table 4:** Type I error rates of five tests based on simulation set 1 for qualitative traits.

Number of Subpopulations	Test	Alpha	Un-corrected	GC	PC-linear	Biased Perm	PC-nonp
2	SingleVariant	0.01	0.02914	0.00648	0.01124	0.0065	0.0107
		0.001	0.00592	0.00034	0.00094	0.00056	0.00094
	Regional	0.01	0.04066	0.00924	0.01168	0.0103	0.01192
		0.001	0.0093	0.0009	0.00144	0.001	0.00146
10	SingleVariant	0.01	0.0428	0.01106	0.0106	0.00672	0.0103
		0.001	0.01172	0.00124	0.00162	0.00078	0.00138
	Regional	0.01	0.04862	0.00984	0.0105	0.01034	0.01062
		0.001	0.01174	0.00134	0.00128	0.0008	0.0012
20	SingleVariant	0.01	0.02022	0.01244	0.01734	0.02296	0.01072
		0.001	0.00414	0.00138	0.00354	0.00458	0.00132
	Regional	0.01	0.04014	0.01068	0.03468	0.02694	0.01034
		0.001	0.00952	0.00172	0.00746	0.0051	0.00152

**Table 5:** Type I error rates of four tests based on simulation set 2 for quantitative traits.

Model	Test	Alpha	Un-corrected	GC	PC-linear	PC-nonp
1	SingleVariant	0.01	0.0207	0.0135	0.0228	0.0132
		0.001	0.0042	0.0021	0.0048	0.0014
	Regional	0.01	0.1256	0.0121	0.1171	0.0106
		0.001	0.052	0.0024	0.0463	0.0008
2	SingleVariant	0.01	0.019	0.0139	0.011	0.0115
		0.001	0.0048	0.0026	0.0011	0.0011
	Regional	0.01	0.0374	0.0128	0.0112	0.0082
		0.001	0.0102	0.0009	0.0008	0.0006

**Table 6:** Type I error rates of five tests based on simulation set 2 for qualitative traits.

Model	Test	Alpha	Un-corrected	GC	PC-linear	BiasedPerm	PC-nonp
1	SingleVariant	0.01	0.0384	0.0128	0.0409	0.02564	0.007
		0.001	0.0132	0.0022	0.0151	0.00652	0.0005
	Regional	0.01	0.0248	0.0095	0.0288	0.02752	0.0114
		0.001	0.0056	0.0009	0.005	0.00584	0.0008
2	SingleVariant	0.01	0.0453	0.0174	0.0087	0.00514	0.0088
		0.001	0.0182	0.0048	0.0006	0.00032	0.001
	Regional	0.01	0.0366	0.0115	0.0121	0.00684	0.0093
		0.001	0.0078	0.0007	0.0016	0.00046	0.0012
