# Supplementary material for: A Nonparametric Regression Approach to Control for Population Stratification in Rare Variant Association Studies
Source: Sci Rep. 2016 Nov 18;6:37444. doi: 10.1038/srep37444 (PMC5114546; doi:10.1038/srep37444)
Supplement: Supplemental Materials [file srep37444-s1.doc]

**A Nonparametric Regression Approach to Control for Population Stratification in Rare Variant Association Studies**

Qiuying Sha^1^, Kui Zhang^1^, Shuanglin Zhang^1,*^

^1^Department of Mathematical Sciences, Michigan Technological University, Houghton, MI 49931

**Supplemental Materials**

*Details of Generating Genotypes for Spatially Structured Populations*

To generate genotypes of parents, we follow Mathieson and McVean (2012). Suppose that we had a $K_{0}\times K_{0}$ grid and we wished to simulate a sample of $C=cK_{0}^{2}$ individuals, where *c* is the number of individuals in each grid square. We simulated L loci on each of *G* genealogies for a total of $L\times G$ loci. Each genealogy represented an independent genomic region, with no recombination inside each region. We indexed the grid squares by *i, j* and denoted the number of lineages in grid square *i, j* at time *t* by $n_{i,j}^{t}$. We let $s_{i,j}$ represent the number of grid squares adjacent to *i, j* in a Manhattan sense, such that $s_{i,j}\in\{2,3,4\}$.

Then, we started at *t = 0* and repeated the following steps until only one lineage remained.

1. At time *t*, the rate of coalescence within grid square *i, j* and the total rate of coalescence were, respectively,

where we use ‘•’ to represent summation over indices. The rate of migration for each grid square was

$$\mu_{i,j}^{t}=\frac{Mn_{i,j}^{t}s_{i,j}}{2}$$

and the total rate of migration was . The next event occurred at time *t + T*, where . This next event was chosen on the basis of the probability of coalescence and the probability of migration .

2. If the next event was coalescence, it occurred in grid square *i, j* with probability . In this grid square, we chose two lineages uniformly and joined them together. We then returned to step 1 with *t* replaced by $t+\text{ }T$.

3. If the next event was migration, it occurred in grid square *i, j* with probability . In this grid square, we chose one lineage uniformly and moved it uniformly to one of the adjacent grid squares. We returned to step 1 with *t* replaced by $t+\text{ }T$.

Once we had simulated a single instance of the genealogy, we generated genotypes at *L* random loci by sampling *L* nodes from the genealogy with replacement, selecting each node with probability proportional to the length of the branch above that node and setting each individual’s genotype to 0 or 1 at each locus according to whether they were descended from that node or not, such that a genotype of 0 represented an ancestral allele and a genotype of 1 represented a derived allele.


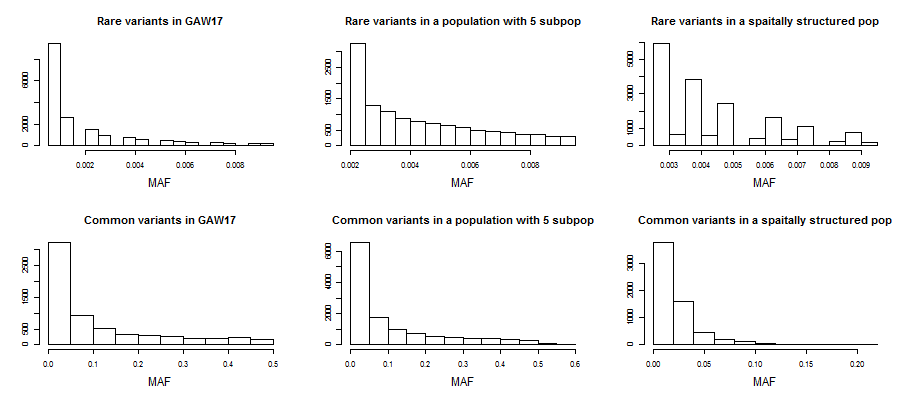


**Figure S1**. The distributions of minor allele frequencies (MAF) of rare variants (common variants) in GAW17 data, in a population with 5 subpopulations, and in a spatially structured population.


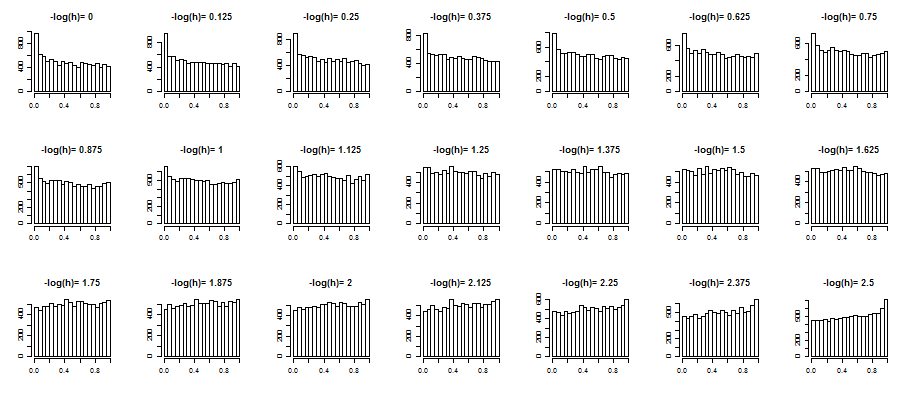


**Figure S2**. 21 histograms for p-values of the proposed test. Each histogram is corresponding to a specific value of smoothing parameter h. This set of simulations is based on a structured population with 5 subpopulations (simulation set 1 with $k_{0}=5$).


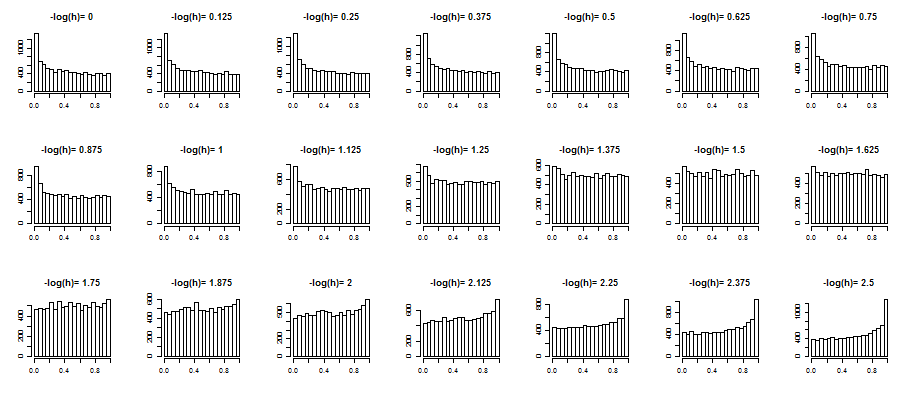


**Figure S3**. 21 histograms for p-values of the proposed test. Each histogram is corresponding to a specific value of smoothing parameter h. This set of simulations is based on a structured population with 10 subpopulations (simulation set 1 with $k_{0}=10$).


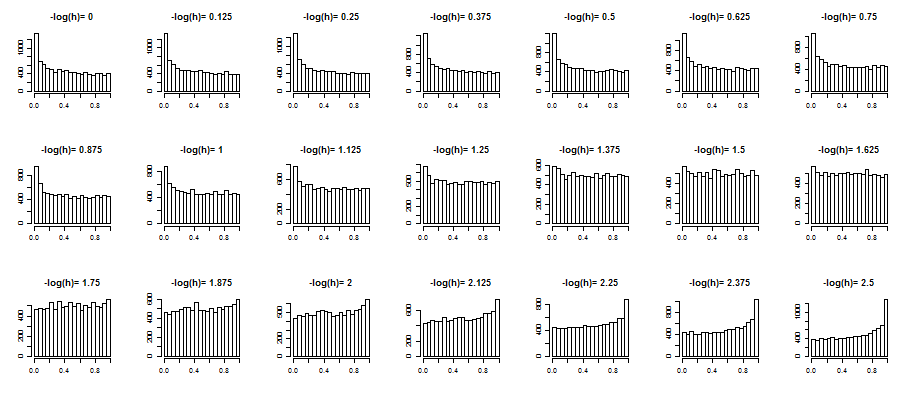


**Figure S4**. 21 histograms for p-values of the proposed test. Each histogram is corresponding to a specific value of smoothing parameter h. This set of simulations is based on a spatially structured population and trait values are generated according to model 1 (simulation set 2 model 1).


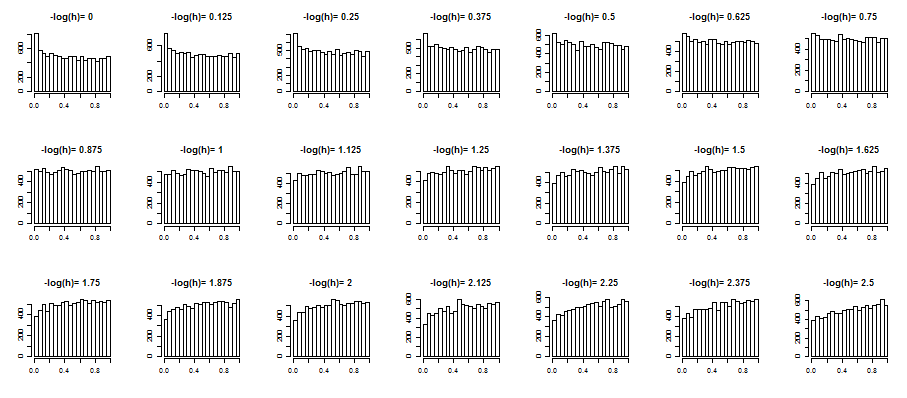


**Figure S5**. 21 histograms for p-values of the proposed test. Each histogram is corresponding to a specific value of smoothing parameter h. This set of simulations is based on a spatially structured population and trait values are generated according to model 2 (simulation set 2 model 2).
